# Genome-Wide Analysis Points to Roles for Extracellular Matrix Remodeling, the Visual Cycle, and Neuronal Development in Myopia

**DOI:** 10.1371/journal.pgen.1003299

**Published:** 2013-02-28

**Authors:** Amy K. Kiefer, Joyce Y. Tung, Chuong B. Do, David A. Hinds, Joanna L. Mountain, Uta Francke, Nicholas Eriksson

**Affiliations:** 23andMe, Mountain View, California, United States of America; Georgia Institute of Technology, United States of America

## Abstract

Myopia, or nearsightedness, is the most common eye disorder, resulting primarily from excess elongation of the eye. The etiology of myopia, although known to be complex, is poorly understood. Here we report the largest ever genome-wide association study (45,771 participants) on myopia in Europeans. We performed a survival analysis on age of myopia onset and identified 22 significant associations (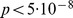), two of which are replications of earlier associations with refractive error. Ten of the 20 novel associations identified replicate in a separate cohort of 8,323 participants who reported if they had developed myopia before age 10. These 22 associations in total explain 2.9% of the variance in myopia age of onset and point toward a number of different mechanisms behind the development of myopia. One association is in the gene *PRSS56*, which has previously been linked to abnormally small eyes; one is in a gene that forms part of the extracellular matrix (*LAMA2*); two are in or near genes involved in the regeneration of 11-cis-retinal (*RGR* and *RDH5*); two are near genes known to be involved in the growth and guidance of retinal ganglion cells (*ZIC2*, *SFRP1*); and five are in or near genes involved in neuronal signaling or development. These novel findings point toward multiple genetic factors involved in the development of myopia and suggest that complex interactions between extracellular matrix remodeling, neuronal development, and visual signals from the retina may underlie the development of myopia in humans.

## Author Summary

The genetic basis of myopia, or nearsightedness, is believed to be complex and affected by multiple genes. Two genetic association studies have each identified a single genetic region associated with myopia in European populations. Here we report the results of the largest ever genetic association study on myopia in over 45,000 people of European ancestry. We identified 22 genetic regions significantly associated with myopia age of onset. Two are replications of the previously identified associations, and 20 are novel. Ten of the novel associations replicate in a small separate cohort. Sixteen of the novel associations are in or near genes implicated in eye development, neuronal development and signaling, the visual cycle of the retina, and general morphology: *BMP3*, *BMP4*, *DLG2*, *DLX1*, *KCNMA1*, *KCNQ5*, *LAMA2*, *LRRC4C*, *PRSS56*, *RBFOX1*, *RDH5*, *RGR*, *SFRP1*, *TJP2*, *ZBTB38*, and *ZIC2*. These findings point to numerous biological pathways involved in the development of myopia and, in particular, suggest that early eye and neuronal development may lead to the eventual development of myopia in humans.

## Introduction

Myopia, or nearsightedness, is the most common eye disorder worldwide. In the United States, an estimated 30–40% of the adult population has clinically relevant myopia (more severe than −1 diopter), and the prevalence has increased markedly in the last 30 years [1], [2]. Myopia is a refractive error that results primarily from increased axial length of the eye [3]. The increased physical length of the eye relative to optical length causes images to be focused in front of the retina, resulting in blurred distance vision.

The etiology of myopia is multifactorial [3]. Briefly, postnatal eye growth is directed by visual stimuli that evoke a signaling cascade within the eye. This cascade is initiated in the retina and passes through the retinal pigment epithelium (RPE) and choroid to guide remodeling of the sclera (the white outer wall of the globe) (cf. [4], [5]). Animal models implicate these visually-guided alterations of the scleral extracellular matrix in the eventual development of myopia [4], [6].

The human eye grows from an average of 17 mm at birth to 21–22 mm in adulthood [7]. By ages 5–6 only about 2% of children are myopic [7]. Although the eye grows only 0.5 mm through puberty [8], the incidence of myopia increases sevenfold during this time [7], peaking between the ages 9–14 [9]. Myopia developed during childhood or early adolescence generally worsens throughout adolescence and then stabilizes by age 20. Compared to myopia that develops in childhood or adolescence, adult onset myopia tends to be less severe [10]–[12]. The majority of myopia cases are primary and nonsyndromic [3]; however, myopia can arise as a complication of other conditions, such as severe prematurity, cataracts, and keratoconus [13], [14], and is sometimes associated with certain connective tissue disorders, such as Stickler syndrome [15].

Although epidemiological studies have implicated numerous environmental factors in the development of myopia, most notably education, outdoor exposure, reading, and near work [3], it is well established that genetics plays a substantial role. Twin and sibling studies have provided heritability estimates that range from 50% to over 90% [16]–[20]. Children of myopic parents tend to have longer eyes and are at higher than average risk of developing myopia in childhood [21]. Segregation analyses suggest that multiple genes are involved in the development of myopia [22], [23]. To date, there have been seven genome-wide association studies (GWAS) on myopia or related phenotypes (pathological myopia, refractive error, and ocular axial length): two in Europeans [24], [25] and five in Asian populations [26]–[30]. Each of these publications has identified a different single association with myopia. In addition there have been several linkage studies (see [3], [31] for reviews) and an exome sequencing study of severe myopia [32].

In contrast to the previous GWAS that used degree of refractive error as a quantitative dependent measure, we analyzed data for 45,771 individuals from the 23andMe database who reported whether they had been diagnosed with nearsightedness, and if so, at what age. We performed a genome-wide survival analysis on age of onset of myopia, discovering 22 genome-wide significant associations with myopia age of onset, 20 of which are novel. Ten of the novel and one of the previously identified associations replicate in a separate (smaller and more coarsely phenotyped) cohort of 8,323 individuals.

## Results/Discussion

Participants reported via web-based questionnaires whether they had been diagnosed with nearsightedness, and if so, at what age. Only those participants who reported onset between five and 30 years of age were included to limit cases of secondary myopia (e.g., myopia due to premature birth or cataracts). Further filtering was performed to limit errors in reporting (see Methods).

All participants were customers of 23andMe and of primarily European ancestry; no pair was related at the level of first cousins or closer. We performed a genome-wide survival analysis using a Cox proportional hazards model on data for 45,771 individuals (“discovery set”). The Cox model assumes that there is an (unknown) baseline probability of developing myopia at every year of age. The model then tests whether each single nucleotide polymorphism (SNP) is associated with a significantly higher or lower probability of developing myopia compared to baseline. The Cox model can be thought of as a generalization of an analysis of myopia age of onset. In contrast to an analysis of age of onset, the Cox model allows for the inclusion of non-myopic controls, resulting in increased statistical power. Analyses controlled for sex and five principal components of genetic ancestry. An additional, non-overlapping set of 8,323 participants who reported on their use of corrective eyewear for nearsightedness before the age of ten were used as a replication set. See Table 1 for characteristics of the two cohorts.

**Table 1:** Cohort statistics.

	Number	% female	Age (SE)	Age of onset (SE)
Discovery, myopic	25,999	45.9	47.7 (15.5)	13.6 (5.8)
Discovery, not myopic	19,772	40.3	49.6 (17.1)	—
Replication, myopic at 10	1,488	45.3	46.7 (14.9)	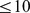
Replication, not myopic at 10	6,835	45.2	53.7 (15.1)	—

Sex, current age, and age of onset for discovery and replication cohorts.

**Figure 1:**
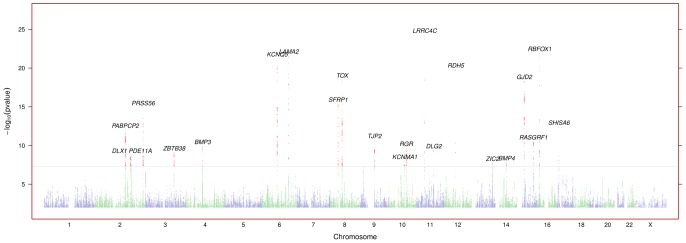
Negative 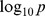-values genome wide for myopia. Regions are named with their postulated candidate gene or genes. 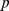-values under 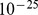 have been cut off (only the *LAMA2* and *LRRC4C* regions are affected). See Figure S1 for plots in each region with a significant association.

Table 2 shows the top SNPs for all 35 genetic regions associated with myopia with a 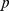-value smaller than 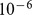. All 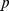-values from the GWAS have been corrected for the inflation factor of GC = 1.167. A total of 22 of the SNPs cross our threshold for genome-wide significance (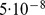, see Figure S1). These 22 include two SNPs previously associated with refractive error in GWAS of European populations: rs524952 near *GJD2* and *ACTC1* and rs28412916 near *RASGRF1* [24], [25], [33]. 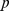-values genome-wide are shown in Figure 1; Figure S2 shows the quantile-quantile plot for the analysis. Table S3 shows all SNPs with 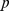-values under 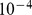.

**Table 2:** Index SNPs for regions with 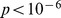.

rsid	chr	Position	Genes	MAF	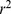	allele	HR (CI)	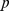 -value	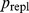
rs12193446	6	129820038	*LAMA2*	0.094	0.991	A/G	0.788 (0.763–0.813)	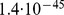	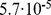
rs1381566	11	40149607	*LRRC4C*	0.181	0.873	T/G	1.149 (1.122–1.176)	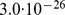	**0.0038**
rs17648524	16	7459683	*RBFOX1*	0.365	0.974	G/C	1.102 (1.082–1.122)	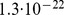	0.36
rs7744813	6	73643289	*KCNQ5*	0.405	0.958	A/C	0.909 (0.893–0.926)	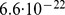	0.15
rs3138142	12	56115585	*RDH5*	0.218	0.831	C/T	0.890 (0.870–0.911)	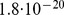	**0.011**
chr8:60178580	8	60178580	*TOX/CA8*	0.358	0.971	C/G	0.914 (0.897–0.931)	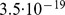	**0.0061**
rs524952	15	35005886	*GOLGA8B/GJD2*	0.469	0.982	T/A	1.089 (1.070–1.108)	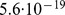	**0.040**
rs2137277	8	40734662	*SFRP1*	0.189	0.922	A/G	0.901 (0.880–0.923)	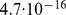	0.46
rs1550094	2	233385396	*PRSS56*	0.305	0.965	A/G	1.087 (1.067–1.107)	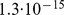	**0.031**
rs2908972	17	11407259	*SHISA6*	0.397	0.969	T/A	1.074 (1.055–1.093)	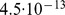	**0.034**
rs17412774	2	146773948	*PABPCP2*	0.450	0.976	A/C	0.933 (0.917–0.950)	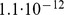	0.067
rs11145746	9	71834380	*TJP2*	0.198	0.887	G/A	1.087 (1.063–1.112)	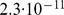	0.87
rs28412916	15	79378167	*RASGRF1*	0.401	0.989	A/C	1.067 (1.048–1.086)	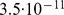	0.08
rs5022942	4	81959966	*BMP3*	0.229	0.991	G/A	1.076 (1.054–1.098)	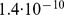	**0.0093**
rs745480	10	85986554	*RGR*	0.473	0.975	C/G	1.063 (1.044–1.081)	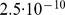	0.095
rs2155413	11	84634790	*DLG2*	0.466	0.997	C/A	1.061 (1.043–1.080)	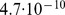	**0.023**
rs13091182	3	141133960	*ZBTB38*	0.333	0.994	G/A	0.940 (0.923–0.958)	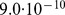	0.31
rs17400325	2	178565913	*PDE11A*	0.050	0.933	T/C	1.144 (1.099–1.190)	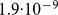	**0.027**
rs17428076	2	172851936	*DLX1*	0.237	0.985	C/G	0.935 (0.916–0.955)	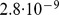	0.53
rs6480859	10	79081948	*KCNMA1*	0.363	0.987	C/T	1.058 (1.039–1.077)	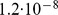	0.40
chr14:54413001	14	54413001	*BMP4*	0.489	0.933	G/C	0.946 (0.929–0.963)	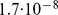	0.21
rs4291789	13	100672921	*ZIC2*	0.326	0.724	C/G	1.069 (1.046–1.092)	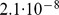	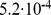
rs10963578	9	18338649	*SH3GL2/ADAMTSL1*	0.200	0.958	G/A	0.936 (0.915–0.957)	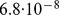	0.15
rs11939401	4	80818417	*ANTXR2*	0.203	0.999	C/T	0.939 (0.919–0.959)	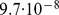	0.13
rs1843303	3	4185124	*SETMAR*	0.303	0.981	T/C	1.055 (1.036–1.075)	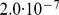	**0.042**
chr11:65348347	11	65348347	*EHBP1L1*	0.017	0.558	G/A	0.770 (0.700–0.846)	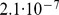	0.54
rs4367880	10	114795256	*TCF7L2*	0.199	0.959	G/C	1.063 (1.040–1.087)	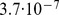	0.57
rs61988414	14	42313443	*LRFN5*	0.168	0.878	A/G	1.071 (1.045–1.097)	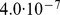	0.84
rs9365619	6	164251746	*QKI*	0.457	0.999	C/A	1.048 (1.031–1.067)	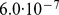	**0.047**
rs4245599	10	60365755	*BICC1*	0.466	0.952	G/A	1.049 (1.031–1.068)	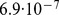	0.44
rs10512441	17	31239645	*MYO1D/TMEM98*	0.203	0.919	C/T	1.062 (1.039–1.085)	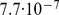	0.39
rs9902755	17	47220726	*B4GALNT2*	0.470	0.668	C/T	1.059 (1.037–1.081)	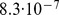	0.91
rs6702767	1	200844547	*GPR25*	0.485	0.982	G/A	1.048 (1.030–1.066)	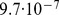	0.40
chr17:79585492	17	79585492	*NPLOC4*	0.393	0.604	G/A	1.063 (1.039–1.087)	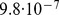	**0.032**
rs6487748	12	9435768	*PZP*	0.491	0.981	A/G	1.048 (1.030–1.066)	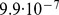	0.42

Index SNPs for regions with (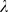-corrected) 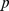-values under 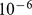. Positions and alleles are given relative to the positive strand of build 37 of the human genome; alleles are listed as major/minor. The listed genes are the postulated candidate gene in each region. 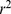 is the estimated imputation accuracy; HR is the hazard ratio per copy of the minor allele; 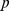-value is the 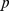-value in the discovery cohort; 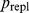 is the 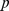-value in the replication cohort. Significant replication 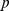-values are bolded.

Of the 22 SNPs significant in the discovery set, 11 were also significant in the replication set (Table 2). Of the 11 SNPs that did not replicate, only two showed different signs between the discovery and replication sets (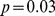). Given these results, and considering that the replication set was much smaller than the discovery set and measured age of onset less exactly, we suspect that much of the lack of replication is due to lack of power.

**Figure 2:**
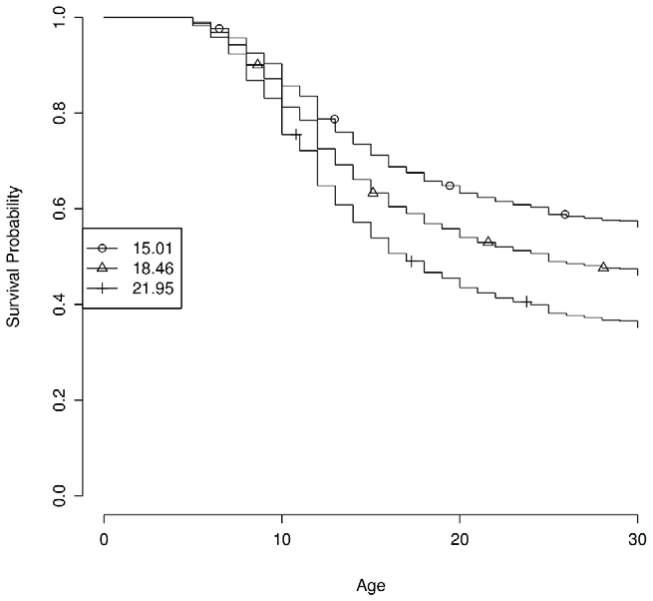
Estimated survival curves by genetic propensity score. The genetic propensity score is computed as the number of risk alleles across the 22 genome-wide significant SNPs. Curves show estimated survival probability (i.e., the probability of not having developed myopia) by age under the fitted Cox model for the 10th, 50th, and 90th percentiles of scores (15.01, 18.46, and 21.95, respectively).

We defined a genetic myopia propensity score as the number of copies of the risk alleles across all 22 SNPs identified via the discovery set. The propensity score showed a strong association with early onset myopia (less than 10 years old) in our replication cohort (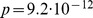, odds ratio 1.075 per risk allele). The top decile of genetic propensity had 1.97 greater odds of developing myopia before the age of 10 than the bottom decile. In a Cox model fit to the discovery set, the propensity score explains 2.9% of the total variance. Note that this estimate may be inflated, as it is calculated on the discovery population. In this model, someone in the 90th percentile of risk (a score of 21.95) is nearly twice as likely to develop myopia by the age of 25 as someone in the 10th percentile of risk (score of 15.01), Figure 2.

Of the 20 novel associations, many lie in or near genes with direct links to processes related to myopia development. Two of them lie in regions associated with myopia in linkage studies: rs1550094 in *PRSS56* (MIM: 609995) [34] and chr14:54413001 near *BMP4* (MIM: 255500) [35]. Two suggestive associations also are in such regions: rs4245599 in *BICC1* (MIM: 612717) [36] and rs9902755 in *B4GALNT2* (MIM: 608474) [37]. Below, we briefly sketch out possible connections between these associations and extracellular matrix (ECM) remodeling, the visual cycle, eye and body growth, retinal neuron development, and general neuronal development or signaling.

### Extracellular Matrix Remodeling

The strongest association is a SNP in an intron of *LAMA2* (laminin, alpha 2 subunit, rs12193446, 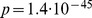, hazard ratio (HR) = 0.79). Laminins are extracellular structural proteins that are integral parts of the ECM. Changes in the composition of the ECM of the sclera have been shown to alter the axial length of the eye [5]. Laminins play a role in the development and maintenance of different eye structures [38], [39]. The laminin alpha 2 chain in particular is found in the extraocular muscles during development [38], and may act as an adhesive substrate and possibly a guidance cue for retinal ganglion cell growth cones [40]. We also found a suggestive association related to laminin (rs11939401, 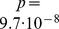, HR = 0.939) approximately 17 kb upstream of *ANTXR2* (anthrax toxin receptor 2). ANTXR2 binds laminin and possibly collagen type IV [41] and thus may also be involved in extracellular matrix remodeling.

### The Visual Cycle

Two of the novel associations are in or near genes involved in the regeneration of 11-cis-retinal, the light sensitive component of photoreceptors, a process commonly referred to as the visual cycle of the retina. These associations are with rs3138142, 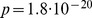, HR = 0.89, in *RDH5* (retinol dehydrogenase 5 (11-cis/9-cis)) and rs745480 (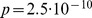, HR = 1.06), a SNP 18 kb upstream of *RGR*, which encodes the retinal G protein-coupled receptor. The SNP rs3138142 is a synonymous change in *RDH5*. It has been linked to *RDH5* expression [42], [43], and it is part of an Nr2f2 (nuclear receptor subfamily 2, group F, member 2) transcription factor binding motif in mouse [44], [45]. Both *RDH5* and *RGR* play crucial roles in the regeneration of 11-cis retinal in the RPE [46]. Mutations in *RDH5* have been linked with fundus albipunctatus, a rare form of congenital stationary night blindness (for a recent review, see [47]) and progressive cone dystrophy [48], and mutations in *RGR* have been linked with autosomal recessive and autosomal dominant retinitis pigmentosa [49], [50].

We also identified an association within another gene that functions in the RPE: rs7744813 (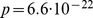, HR = 0.91), a SNP in *KCNQ5* (potassium voltage-gated channel, KQT-like subfamily, member 5). *KCNQ5* encodes a potassium channel found in the RPE and neural retina. These channels are believed to contribute to ion flow across the RPE [51], [52] and to affect the function of cone and rod photoreceptors [52].

### Eye and Body Growth

Five of our associations show possible links to eye or overall morphology. The first is a missense mutation in *PRSS56* (A224T, rs1550094, 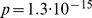, HR = 1.09). Other mutations in *PRSS56* have been shown to cause strikingly small eyes with severe decreases in axial length [53]–[55]. Two other associated SNPs are near genes that encode bone morphogenetic proteins: chr14:54413001 (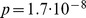, HR = 0.95) near *BMP4* (bone morphogenic protein-4), and rs5022942 (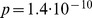, HR = 1.08) in *BMP3* (bone morphogenic protein-3). Inherited *BMP4* mutations have been associated with syndromic microphthalmia and various eye, brain and digital malformations [56], [57]. Although *BMP3* is primarily known for its role in bone development (e.g., it is linked to skeletal defects in humans and skull shapes in dogs [58], [59]), it was found to be uniquely expressed in keratocytes, specialized mesenchymal cells that are important for development of the cornea by producing and maintaining the extracellular matrix of the corneal stroma [60]. One associated SNP, rs13091182 (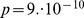, HR = 0.94), in *ZBTB38* (zinc finger and BTB domain-containing protein 38), is in linkage disequilibrium (LD) with a SNP previously associated with height (rs6763931; 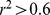) [61]. The final SNP with a link to morphology is rs17428076 (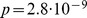, HR = 0.94), near *DLX1* (homo sapiens distal-less homeobox 1). Disruption of *DLX1* has been shown to result in poor optic cup regeneration in planarians and small eyes in mice [62], [63].

### Retinal Ganglion Cell Projections

Two of the novel associations are near genes that affect the outgrowth of retinal ganglion neurons during development. The first is rs4291789 (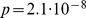, HR = 1.07), which lies 34 kb downstream of *ZIC2* (Zic family member 2). ZIC2 regulates two independent parts of ipsilateral retinal ganglion cell development: axon repulsion at the optic chiasm midline [64], [65], and organization of the axonal projections at their final targets in the brain [66].

The second, rs2137277 (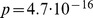, HR = 0.90), is a variant in *ZMAT4* (zinc finger, matrin-type 4). *ZMAT4* has no known link to vision, but this variant also lies 385 kb downstream of *SFRP1* (secreted frizzled-related protein 1). *SFRP1* is involved in the differentiation of the optic cup from the neural retina [67], retinal neurogenesis [68], the development and function of photoreceptor cells [69], [70], and the growth of retinal ganglion cells [71].

### Neuronal Signaling and Development

Finally, we found five associations with SNPs in genes involved in neuronal development and signaling, but without a known role in vision development or the vision cycle: in *KCNMA1* (potassium large conductance calcium-activated channel, subfamily M, alpha member 1; rs6480859, 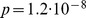, HR = 1.06); in *RBFOX1* (RNA binding protein, fox-1 homolog; rs17648524, 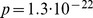, HR = 1.10); in *LRRC4C*, leucine rich repeating region containing 4C, also known as *NGL-1* (rs1381566, 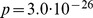, HR = 1.15); in *DLG2* (discs, large homolog 2; rs2155413, 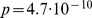, HR = 1.06); and in *TJP2* (tight junction protein 2; rs11145746, 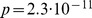, HR = 1.09).

*KCNMA1* encodes the pore-forming alpha subunit of a MaxiK channel, a family of large conductance, voltage and calcium-sensitive potassium channels involved in the control of smooth muscle and neuronal excitation. *RBFOX1* belongs to a family of RNA binding proteins that regulates the alternative splicing of several neuronal transcripts implicated in neuronal development and maturation [72]. *LRRC4C* encodes a binding partner for netrin G1 and promotes the outgrowth of thalamocortical axons [73]. *DLG2* plays a critical role in the formation and regulation of protein scaffolding at postsynaptic sites [74]. *TJP2* has been linked with hearing loss: its duplication and subsequent overexpression are found in adult-onset progressive nonsyndromic hearing loss [75].

### Conclusion

This study represents the largest GWAS on myopia in Europeans to date. This cohort of 45,771 individuals led to the discovery of 20 novel associations as well as replication of the two previously reported associations in Europeans. Ten of these novel associations replicate in our much smaller replication set of 8,323 individuals. In contrast to the earlier studies that used refractive error as a quantitative outcome, we used a Cox proportional hazards model with age of onset of myopia as our major endpoint. This model yielded greater statistical power than a simple case-control study of myopia. Of the 22 significant SNPs found using this model, all but two had smaller 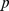-values when a hazards model was employed, and only 20 would be genome-wide significant using a case-control analysis on the same dataset (Table S1).

The proportional hazards model assumes that the effect of each SNP on myopia risk does not vary by age. When we tested the validity of this assumption for the 22 significant SNPs, only the one in *LAMA2* (rs12193446) showed evidence of different effects at different ages (Table S2). While this violation should not lead to overly small 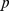-values for this SNP in the GWAS, it does make risk prediction based on these results less straightforward. For example, rs12193446 shows a large effect on myopia hazard at an early age, peaking around 11 years, and then a null or even negative effect on hazard at older ages (Figure S3). This age dependent hazard suggests that different biological processes may affect the development of myopia at different ages.

Our findings further suggest that there may be somewhat different genetic factors underlying myopia age of onset and refractive error. Because adult onset myopia tends to be less severe than myopia developed in childhood or adolescence [10]–[12], age of onset is likely correlated with refractive error, but it is not known how strongly. Many of the associations with myopia age of onset that we found are stronger than the two previously detected associations with refractive error (near *GJD2* and near *RASGRF1*). Notably, the latter association, near *RASGRF1*, also failed to replicate in a recent meta-analysis [33]. The fact that many of our associations with strong effects on age of onset have not shown up in previous refractive error GWAS implies that some genetic factors may affect the age of onset independent of eventual severity, and that the strength of different genetic associations with myopia may depend on the specific phenotype under study.

We also note that our phenotype was based on participants' reports rather than clinical assessments. Although in theory errors in recall could have affected our results, we expect that the vast majority of people are able to recall when they first wore glasses with at most a few years of error. In fact, a subset of those eligible to be part of our discovery cohort provided age of myopia diagnosis in two independent places (see Methods for details). Out of 1,463 people who reported age of diagnosis in both surveys and met our inclusion criteria (European ancestry, age at diagnosis between five and 30 and less than current age), 96.0% reported ages that differed by at most three years and 97.8% by at most five years.

The five associations previously reported in pathological myopia or refractive error GWAS in Asian populations [26]–[30] show no overlap with the significant or suggestive regions found here. Nor did we find an association with the *ZNF644* locus that was identified as the site of high-penetrance, autosomal dominant mutations in Han Chinese families with apparent monogenic inheritance of high-grade myopia [32]. This lack of overlap could result from different genetic factors being involved in myopia across populations. It has been suggested that pathological myopia, which represents less than 2% of cases in the United States [1], has different underlying genetic factors than non-pathological myopia [31].

Our identification of 20 novel genetic associations suggests several novel genetic pathways in the development of human myopia. These findings augment existing research on the development of myopia, which to date has been studied primarily in animal models of artificially induced myopia. Some of the associations are consistent with the current view, based largely on animal models, that a visually-triggered signaling cascade from the retina ultimately guides the scleral remodeling that leads to eye growth, and that the RPE plays a key role in this process [4]. A number of the novel associations point to the potential importance of early neuronal development in the eventual development of myopia, particularly the growth and topographical organization of retinal ganglion cells. These associations suggest that early neuronal development may also contribute to future refractive errors. We expect that these findings will drive new research into the complex etiology of myopia.

## Methods

### Human Subjects

All participants were drawn from the customer base of 23andMe, Inc., a consumer genetics company. This cohort has been described in detail previously [76], [77]. Participants provided informed consent and participated in the research online, under a protocol approved by the external AAHRPP-accredited IRB, Ethical & Independent Review Services (E&I Review).

### Phenotype Data

Participants in the discovery cohort were asked online as part of a medical history questionnaire or an eyesight questionnaire: “Have you ever been diagnosed by a doctor with any of the following vision conditions?: Nearsightedness (near objects are clear, far objects are blurry) (Yes/No/I don't know)”. If they answered “yes”, they were asked, “At what age were you first diagnosed with nearsightedness (near objects are clear, far objects are blurry)? Your best guess is fine.” Those reporting an age of onset either greater than their current age or outside of the range 5–30 were removed from analysis. All participants also reported current age. A total of 4,758 participants reporting age of onset outside of 5–30 and 87 reporting age of onset in the future were removed.

To limit errors in reporting, we excluded from the discovery cohort those who provided discordant answers in the medical history and eyesight questionnaires. We defined discordance as a disagreement in diagnosis or a difference in more than 5 years in age of onset. A total of 92 people with discordant age of onset and 276 with discordant diagnosis were removed. Many of these people would have been filtered out by our other restrictions: only 32 of the 92 with discordant ages of onset would not have been removed for other reasons (mostly because their stated age of onset was not between 5–30), and only 139 of the 276 with discordant diagnoses. These 32 and 139 are out of 1,463 and 2,845 eligible people, respectively, leading us to estimates of 97.8% and 95.1% concordance in age of onset and myopia diagnosis (after the filters mentioned above were applied).

The replication cohort consisted of 8,323 23andMe customers who were not part of the discovery cohort and were not closely related (at 700 cM or greater IBD) to each other or to anyone in the discovery cohort. They provided information on myopia age of onset in one of two ways. 5,265 answered a single question: “Did you wear glasses or other corrective eyewear for nearsightedness before the age of 10? (Yes/No/I'm not sure).” The other 3,058 provided age of onset in the same manner as the discovery cohort. Note that these 3,058 were people that would have been eligible for the discovery cohort, however, they provided data in the time in between our analysis of the discovery and replication cohorts. Their data was converted to the same binary scale as the first group.

### Genotyping and Imputation

Participants were genotyped and additional SNP genotypes were imputed against the August 2010 release of the 1000 genomes data as described previously [78]. Briefly, they were genotyped on at least one of three genotyping platforms, two based on the Illumina HumanHap550+ BeadChip, the third based on the Illumina Human OmniExpress+ BeadChip. The platforms included assays for 586,916, 584,942, and 1,008,948 SNPs respectively. Genotypes for a total of 11,914,767 SNPs were imputed in batches of roughly 10,000 individuals, grouped by genotyping platform. Of these, 7,087,609 met our criteria of 0.005 minor allele frequency, average 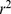 across batches of at least 0.5, and minimum 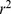 across batches of at least 0.3. (The minimum 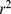 requirement was added to filter out SNPs that imputed poorly in the batches consisting of the less dense platform.)

### Statistical Analysis

In order to minimize population substructure while maximizing statistical power, the study was limited to individuals with European ancestry. Ancestry was inferred from the genome-wide genotype data, and principal component analysis was performed as in [76], [79]. The combined discovery and replication populations were filtered by relatedness to remove participants at a first cousin or closer relationship. More precisely, no two participants shared more than 700 cM of DNA identical by descent (IBD, approximately the lower end of sharing between a pair of first cousins). IBD was calculated using the methods described in [80].

For the survival analysis, let the hazard function 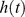 be the rate of developing myopia at time 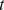. Then the Cox proportional hazards model is
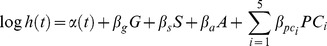
for an arbitrary baseline hazard function 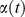 and covariates 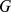 (genotype), 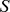 (sex), 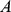 (age), and 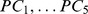 (projections onto principal components). 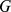 was coded as a dosage from 0–2 as the estimated number of minor alleles present.

For each SNP, we fit a Cox proportional hazards model using R [81] and computed a p-value using a likelihood ratio test for the genotype term. All SNPs with 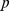-values under 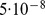 after genomic control correction were considered genome-wide significant. The hazard ratio (HR) reported throughout can be interpreted as the multiplicative change in the rate of onset of myopia per copy of the minor allele (e.g., 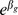). To test the proportional hazards assumption, we tested for independence of the scaled Schoenfeld residuals for each significant SNP and time using cox.zph (Table S2). Replication 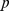-values in Table 2 are one-sided 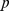-values from a likelihood ratio test for a logistic regression model controlling for age, sex, and five principal components.

For Figure 2, we computed a myopia propensity score for each individual as the (estimated) number of risk alleles among the 22 genome-wide significant SNPs. We then fit a Cox model including that score, sex, and five principal components. To estimate proportion variance explained for this model, we used a pseudo-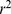 using likelihoods (similar to the Nagelkerke pseudo 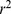 for logistic regression). That is, we calculated the variance explained as
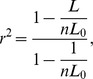
where 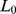 is the null likelihood and 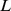 the likelihood for the full model. This is one of several methods used to compute variance explained for Cox proportional hazards models [82].

## Supporting Information

Figure S1Region plots for genome-wide significant associations Colors depict the squared correlation (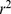) of each SNP with the most associated SNP (shown in purple). Gray indicates SNPs for which 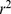 information was missing.(PDF)

Figure S2Quantile-quantile plot for myopia survival analysis Actual (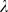-corrected) 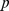-values versus the null.(PDF)

Figure S3Smoothed log-hazard ratios as a function of age for four SNPs In each plot, the straight line shows the estimated log-hazard ratio (beta) for each SNP in the proportional hazards model. The solid curve is a spline fit to beta estimated at different ages; the dotted curves are approximate 95% confidence intervals. The 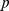-value in each caption is the result of a test of the proportional hazards assumption. The sign of all coefficients has been made positive for ease of comparison (so (a), (c), and (d) are flipped relative to the main text). Note that among the examples here, only (a) shows evidence of deviation from the proportional hazards assumption after correction for 22 tests.(PDF)

Table S1

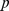
-values for survival and case-control analyses. 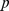-values for SNPs in the survival analysis used in the paper as well as in a case-control logistic regression on the same set of individuals. The survival analysis gives a smaller 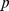-value for 30 of 35 SNPs and has 22 genome-wide significant (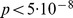) as compared to 20 for the case-control. 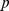-values in both cases are adjusted for the genomic control inflation factor of 1.16.(PDF)

Table S2Tests of deviation from the proportional hazards assumption. 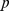-values for significant SNPs for deviation from the proportional hazards assumption in the Cox model. For each SNP, we fit a Cox proportional hazards model including the SNP, sex, and five principal components as predictors, and then tested for independence of the scaled Schoenfeld residuals with time. Only one SNP deviates significantly from this assumption after correction for 22 tests. Plots for four example SNPs are shown in Figure S3.(PDF)

Table S3Statistics for all SNPs with 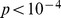. All 6,141 SNPs with (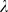-corrected) 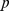-values under 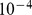 in the discovery cohort. Positions and alleles are given relative to the positive strand of build 37 of the human genome; alleles are listed as major/minor. The gene column shows the position of the SNP in context of the nearest genes. The SNP position is within the brackets, and the number of dashes gives approximate 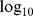 distances. The MAF is the minor allele frequency in Europeans, 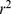 is the estimated imputation accuracy, HR is the hazard ratio per copy of the minor allele, and 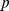-value is the 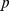-value in the discovery cohort.(XLS)
