# Supplementary material for: Genome-Wide Analysis Points to Roles for Extracellular Matrix Remodeling, the Visual Cycle, and Neuronal Development in Myopia
Source: PLoS Genet. 2013 Feb 28;9(2):e1003299. doi: 10.1371/journal.pgen.1003299 (PMC3585144; doi:10.1371/journal.pgen.1003299)
Supplement: Figure S1 — Region plots for genome-wide significant associations Colors depict the squared correlation () of each SNP with the most associated SNP (shown in purple). Gray indicates SNPs for which information was missing. (PDF) [file pgen.1003299.s001.pdf]

Genome-wide analysis points to roles for extracellular matrix remodeling, the visual cycle, and neuronal development in myopia

Kiefer, Tung, Do, Hinds, Mountain, Francke, Eriksson

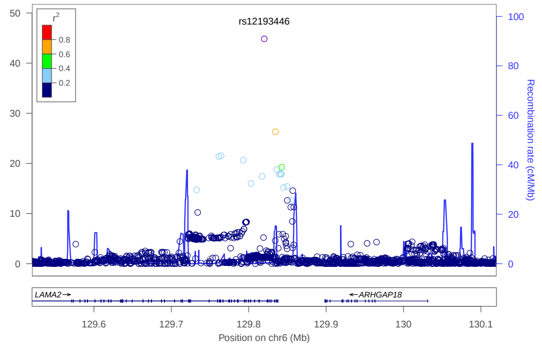

(a) *LAMA2*

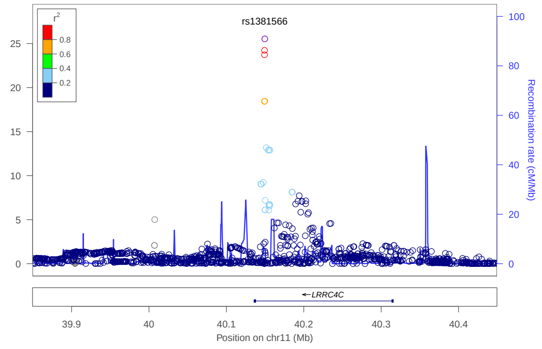

(b) *LRRC4C*

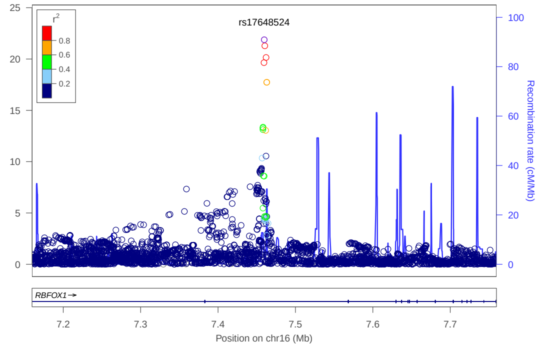

(c) *RBFOX1*

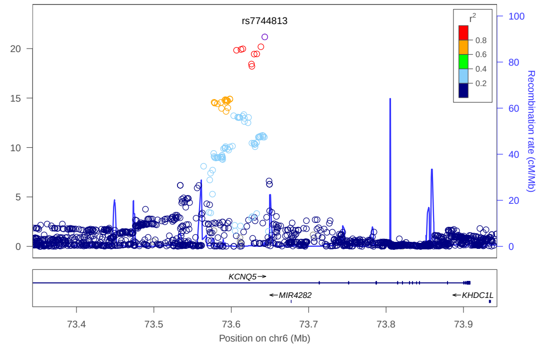

(d) *KCNQ5*

Figure S1: **Region plots for genome-wide significant associations** Colors depict the squared correlation (r^2) of each SNP with the most associated SNP (shown in purple). Gray indicates SNPs for which r^2 information was missing.

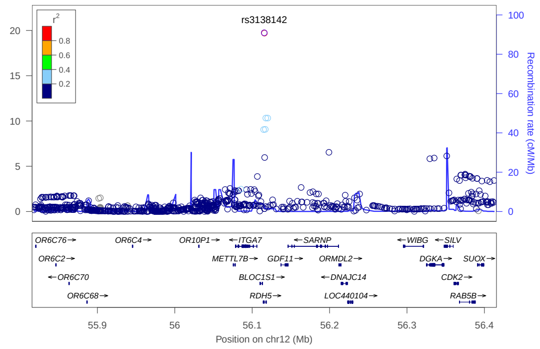

(e) *RDH5*

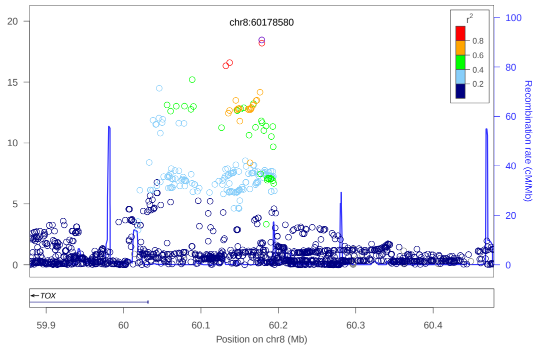

(f) *TOX/CA8*

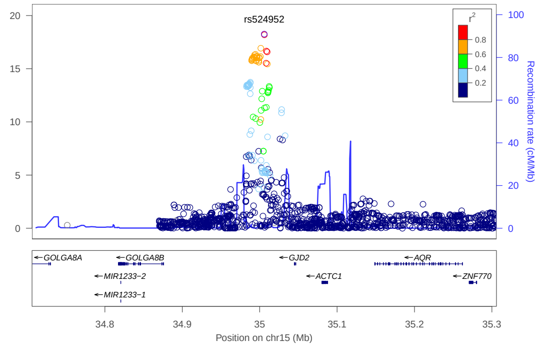

(g) *GOLGA8B/GJD2*

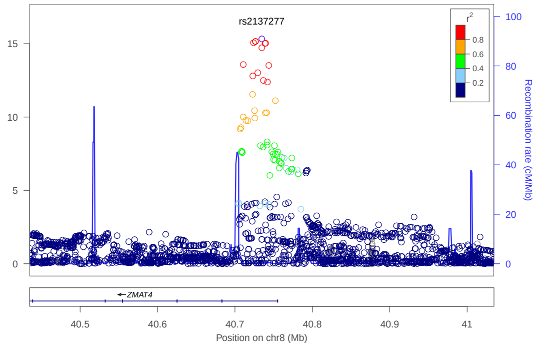

(h) *SFRP1*

Figure S1: **Region plots for genome-wide significant associations** Colors depict the squared correlation (r^2) of each SNP with the most associated SNP (shown in purple). Gray indicates SNPs for which r^2 information was missing.

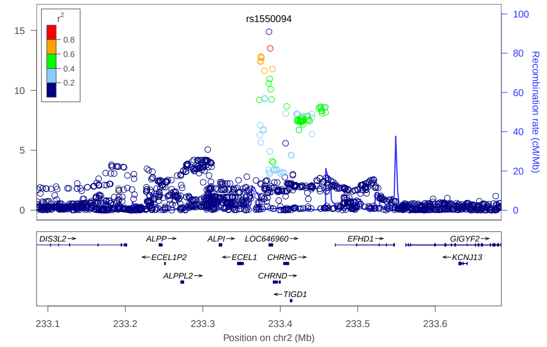

(i) *PRSS56*

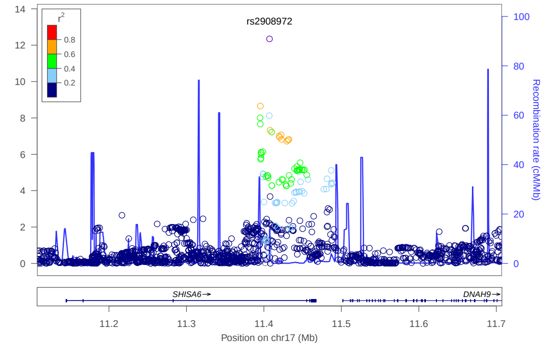

(j) *SHISA6*

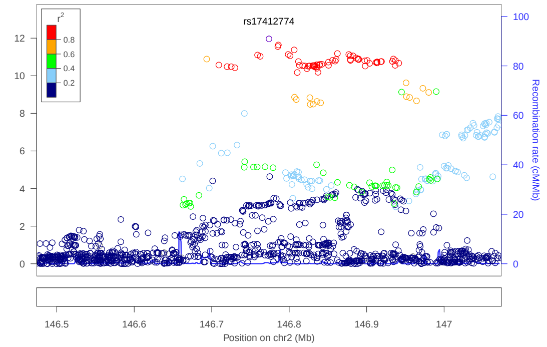

(k) *PABPCP2*

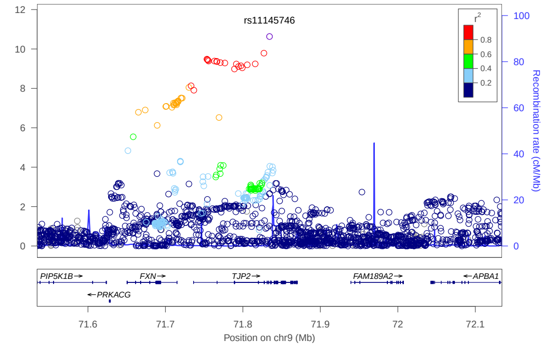

(l) *TJP2*

Figure S1: **Region plots for genome-wide significant associations** Colors depict the squared correlation (r^2) of each SNP with the most associated SNP (shown in purple). Gray indicates SNPs for which r^2 information was missing.

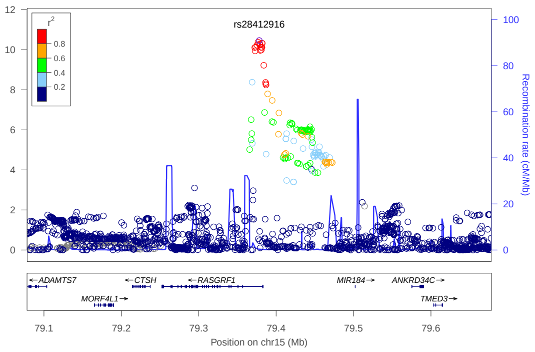

(m) *RASGRF1*

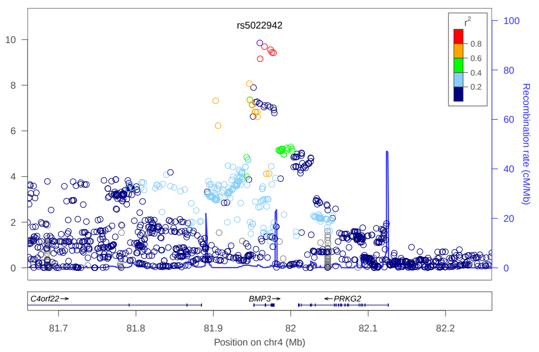

(n) *BMP3*

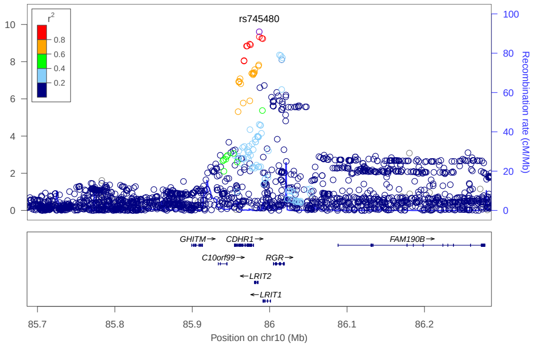

(o) *RGR*

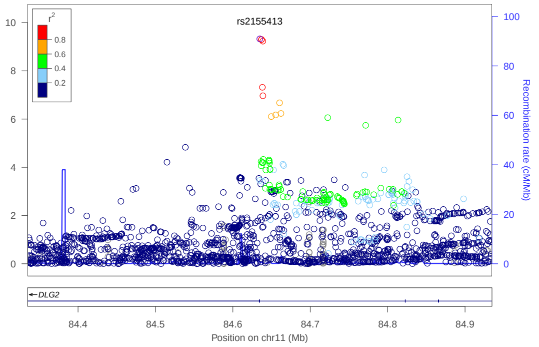

(p) *DLG2*

Figure S1: **Region plots for genome-wide significant associations** Colors depict the squared correlation (r^2) of each SNP with the most associated SNP (shown in purple). Gray indicates SNPs for which r^2 information was missing.

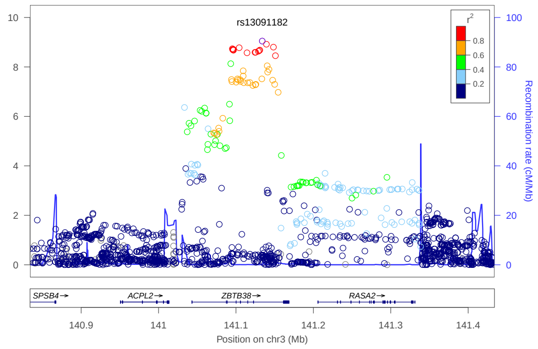

(q) *ZBTB38*

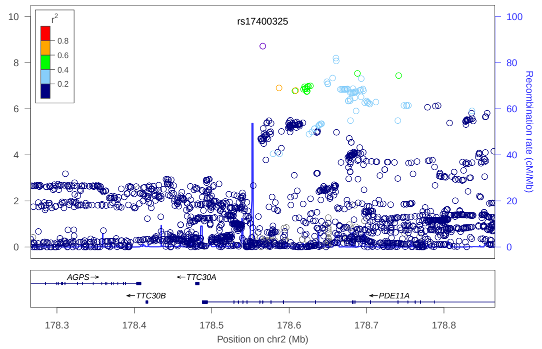

(r) *PDE11A*

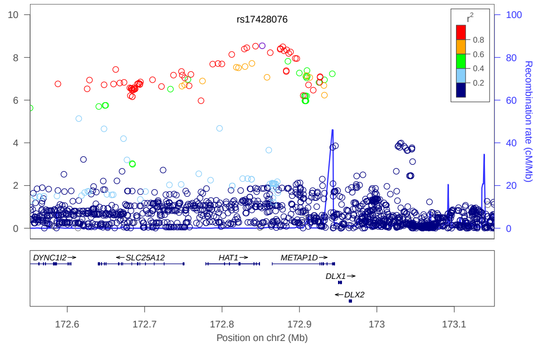

(s) *DLX1*

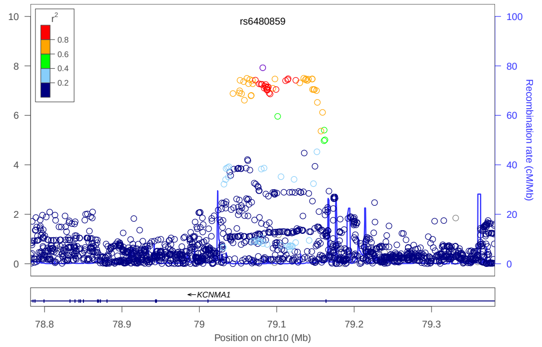

(t) *KCNMA1*

Figure S1: **Region plots for genome-wide significant associations** Colors depict the squared correlation (r^2) of each SNP with the most associated SNP (shown in purple). Gray indicates SNPs for which r^2 information was missing.

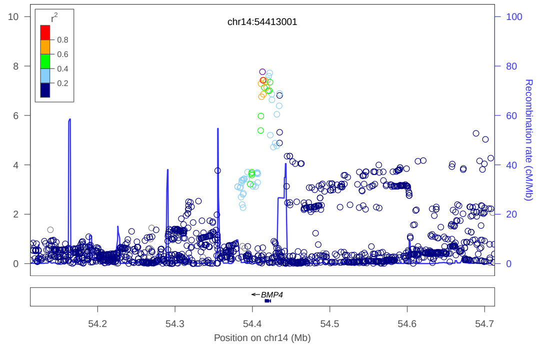

(u) *BMP4*

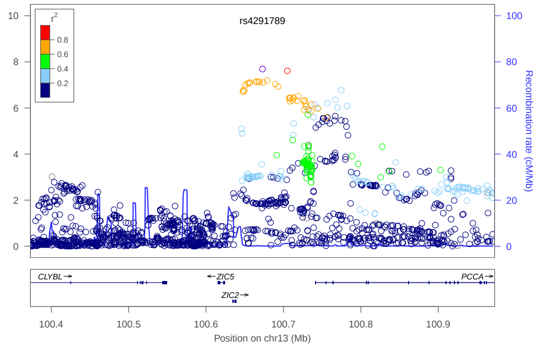

(v) *ZIC2*

Figure S1: **Region plots for genome-wide significant associations** Colors depict the squared correlation (r^2) of each SNP with the most associated SNP (shown in purple). Gray indicates SNPs for which r^2 information was missing.
