# Supplementary material for: Genome-Wide Analysis Points to Roles for Extracellular Matrix Remodeling, the Visual Cycle, and Neuronal Development in Myopia
Source: PLoS Genet. 2013 Feb 28;9(2):e1003299. doi: 10.1371/journal.pgen.1003299 (PMC3585144; doi:10.1371/journal.pgen.1003299)
Supplement: Figure S2 — Quantile-quantile plot for myopia survival analysis Actual (-corrected) -values versus the null. (PDF) [file pgen.1003299.s002.pdf]

Genome-wide analysis points to roles for extracellular matrix remodeling, the visual cycle, and neuronal development in myopia

Kiefer, Tung, Do, Hinds, Mountain, Francke, Eriksson

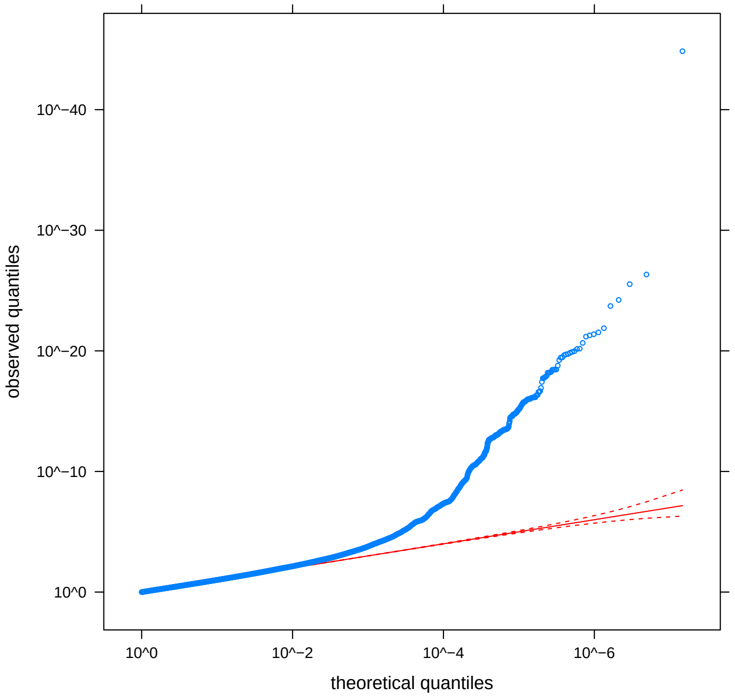

Figure S2: **Quantile-quantile plot for myopia survival analysis** Actual (λ-corrected) p-values versus the null.
