# Supplementary material for: Genome-Wide Analysis Points to Roles for Extracellular Matrix Remodeling, the Visual Cycle, and Neuronal Development in Myopia
Source: PLoS Genet. 2013 Feb 28;9(2):e1003299. doi: 10.1371/journal.pgen.1003299 (PMC3585144; doi:10.1371/journal.pgen.1003299)
Supplement: Figure S3 — Smoothed log-hazard ratios as a function of age for four SNPs In each plot, the straight line shows the estimated log-hazard ratio (beta) for each SNP in the proportional hazards model. The solid curve is a spline fit to beta estimated at different ages; the dotted curves are approximate 95% confidence intervals. The -value in each caption is the result of a test of the proportional hazards assumption. The sign of all coefficients has been made positive for ease of comparison (so (a), (c), and (d) are flipped relative to the main text). Note that among the examples here, only (a) shows evidence of deviation from the proportional hazards assumption after correction for 22 tests. (PDF) [file pgen.1003299.s003.pdf]

Genome-wide analysis points to roles for extracellular matrix remodeling, the visual cycle, and neuronal development in myopia

Kiefer, Tung, Do, Hinds, Mountain, Francke, Eriksson

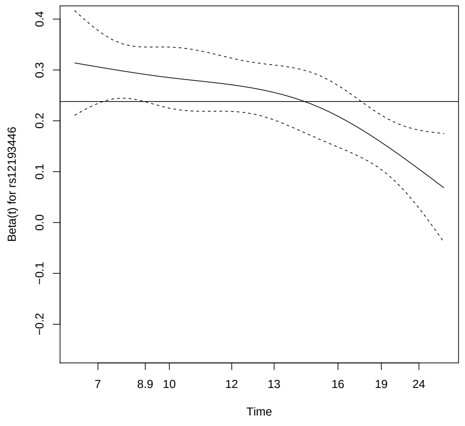

(a) rs12193446, $p = 6.3 \cdot 10^{-5}$

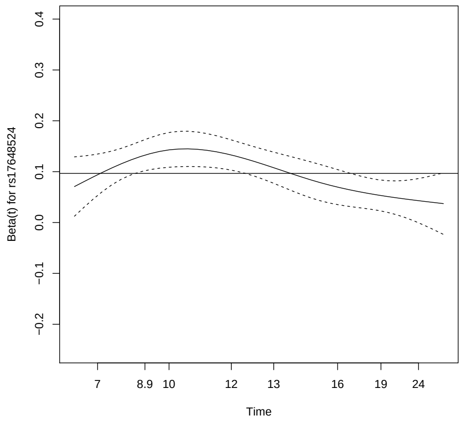

(b) rs17648524, $p = 0.0073$

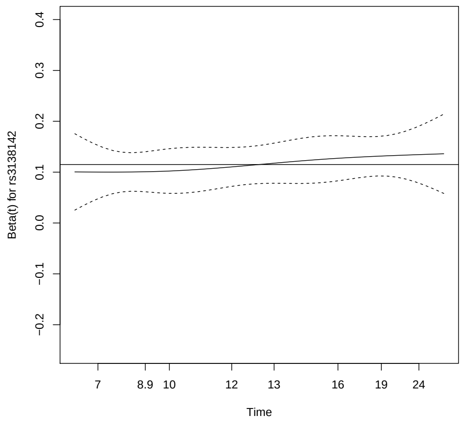

(c) rs3138142, $p = 0.27$

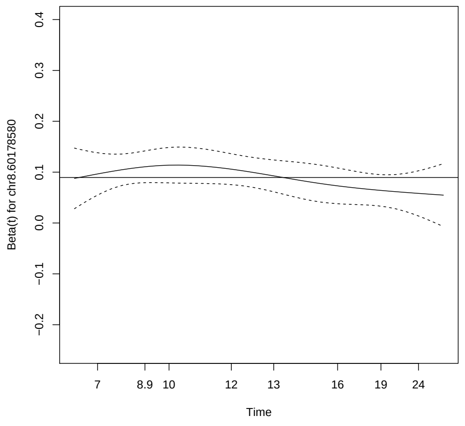

(d) chr8:60178580, $p = 0.081$

Figure S3: **Smoothed log-hazard ratios as a function of age for four SNPs** In each plot, the straight line shows the estimated log-hazard ratio (beta) for each SNP in the proportional hazards model. The solid curve is a spline fit to beta estimated at different ages; the dotted curves are approximate 95% confidence intervals. The p-value in each caption is the result of a test of the proportional hazards assumption. The sign of all coefficients has been made positive for ease of comparison (so (a), (c), and (d) are flipped relative to the main text). Note that among the examples here, only (a) shows evidence of deviation from the proportional hazards assumption after correction for 22 tests.
