# Supplementary material for: Genome-Wide Analysis Points to Roles for Extracellular Matrix Remodeling, the Visual Cycle, and Neuronal Development in Myopia
Source: PLoS Genet. 2013 Feb 28;9(2):e1003299. doi: 10.1371/journal.pgen.1003299 (PMC3585144; doi:10.1371/journal.pgen.1003299)
Supplement: Table S1 — -values for survival and case-control analyses. -values for SNPs in the survival analysis used in the paper as well as in a case-control logistic regression on the same set of individuals. The survival analysis gives a smaller -value for 30 of 35 SNPs and has 22 genome-wide significant () as compared to 20 for the case-control. -values in both cases are adjusted for the genomic control inflation factor of 1.16. (PDF) [file pgen.1003299.s004.pdf]

Genome-wide analysis points to roles for extracellular matrix remodeling, the visual cycle, and neuronal development in myopia

Kiefer, Tung, Do, Hinds, Mountain, Francke, Eriksson

Table S1: **p-values for survival and case-control analyses**

SNP	p (survival)	p (case-control)
rs12193446	$1.4 \cdot 10^{-45}$	$1.29 \cdot 10^{-38}$
rs1381566	$3 \cdot 10^{-26}$	$1.18 \cdot 10^{-24}$
rs17648524	$1.3 \cdot 10^{-22}$	$3.02 \cdot 10^{-19}$
rs7744813	$6.6 \cdot 10^{-22}$	$2.15 \cdot 10^{-19}$
rs3138142	$1.8 \cdot 10^{-20}$	$1.20 \cdot 10^{-20}$
chr8:60178580	$3.5 \cdot 10^{-19}$	$6.53 \cdot 10^{-17}$
rs524952	$5.6 \cdot 10^{-19}$	$1.62 \cdot 10^{-15}$
rs2137277	$4.7 \cdot 10^{-16}$	$3.49 \cdot 10^{-14}$
rs1550094	$1.3 \cdot 10^{-15}$	$4.54 \cdot 10^{-13}$
rs2908972	$4.5 \cdot 10^{-13}$	$4.66 \cdot 10^{-11}$
rs17412774	$1.1 \cdot 10^{-12}$	$1.01 \cdot 10^{-12}$
rs11145746	$2.3 \cdot 10^{-11}$	$5.87 \cdot 10^{-10}$
rs28412916	$3.5 \cdot 10^{-11}$	$2.75 \cdot 10^{-10}$
rs5022942	$1.4 \cdot 10^{-10}$	$6.41 \cdot 10^{-10}$
rs745480	$2.5 \cdot 10^{-10}$	$2.20 \cdot 10^{-8}$
rs2155413	$4.7 \cdot 10^{-10}$	$9.32 \cdot 10^{-9}$
rs13091182	$9 \cdot 10^{-10}$	$7.25 \cdot 10^{-9}$
rs17400325	$1.9 \cdot 10^{-9}$	$3.83 \cdot 10^{-8}$
rs17428076	$2.8 \cdot 10^{-9}$	$1.35 \cdot 10^{-7}$
rs6480859	$1.2 \cdot 10^{-8}$	$1.34 \cdot 10^{-7}$
chr14:54413001	$1.7 \cdot 10^{-8}$	$2.67 \cdot 10^{-7}$
rs4291789	$2.1 \cdot 10^{-8}$	$2.93 \cdot 10^{-8}$
rs10963578	$6.8 \cdot 10^{-8}$	$2.71 \cdot 10^{-8}$
rs11939401	$9.7 \cdot 10^{-8}$	$5.03 \cdot 10^{-8}$
rs1843303	$2 \cdot 10^{-7}$	$1.50 \cdot 10^{-6}$
chr11:65348347	$2.1 \cdot 10^{-7}$	$2.06 \cdot 10^{-6}$
rs4367880	$3.7 \cdot 10^{-7}$	$3.33 \cdot 10^{-7}$
rs61988414	$4 \cdot 10^{-7}$	$5.49 \cdot 10^{-6}$
rs9365619	$6 \cdot 10^{-7}$	$6.48 \cdot 10^{-7}$
rs4245599	$6.9 \cdot 10^{-7}$	$1.92 \cdot 10^{-6}$
rs10512441	$7.7 \cdot 10^{-7}$	$2.62 \cdot 10^{-6}$
rs9902755	$8.3 \cdot 10^{-7}$	$5.51 \cdot 10^{-6}$
rs6702767	$9.7 \cdot 10^{-7}$	$1.77 \cdot 10^{-6}$
chr17:79585492	$9.8 \cdot 10^{-7}$	$5.80 \cdot 10^{-6}$
rs6487748	$9.9 \cdot 10^{-7}$	$2.76 \cdot 10^{-6}$

p-values for SNPs in the survival analysis used in the paper as well as in a case-control logistic regression on the same set of individuals. The survival analysis gives a smaller p-value for 30 of 35 SNPs and has 22 genome-wide significant ($p < 5 \cdot 10^{-8}$) as compared to 20 for the case-control. p-values in both cases are adjusted for the genomic control inflation factor of 1.16.
