# Supplementary material for: Genome-Wide Analysis Points to Roles for Extracellular Matrix Remodeling, the Visual Cycle, and Neuronal Development in Myopia
Source: PLoS Genet. 2013 Feb 28;9(2):e1003299. doi: 10.1371/journal.pgen.1003299 (PMC3585144; doi:10.1371/journal.pgen.1003299)
Supplement: Table S2 — Tests of deviation from the proportional hazards assumption. -values for significant SNPs for deviation from the proportional hazards assumption in the Cox model. For each SNP, we fit a Cox proportional hazards model including the SNP, sex, and five principal components as predictors, and then tested for independence of the scaled Schoenfeld residuals with time. Only one SNP deviates significantly from this assumption after correction for 22 tests. Plots for four example SNPs are shown in Figure S3. (PDF) [file pgen.1003299.s005.pdf]

Genome-wide analysis points to roles for extracellular matrix remodeling, the visual cycle, and neuronal development in myopia

Kiefer, Tung, Do, Hinds, Mountain, Francke, Eriksson

Table S2: **Tests of deviation from the proportional hazards assumption**

SNP	p-value
**rs12193446**	$6.3 \cdot 10^{-5}$
rs1381566	0.68
rs17648524	0.0073
rs7744813	0.19
rs3138142	0.27
chr8.60178580	0.081
rs524952	0.011
rs2137277	0.14
rs1550094	0.0092
rs2908972	0.010
rs17412774	0.40
rs11145746	0.051
rs28412916	0.77
rs5022942	0.33
rs745480	0.017
rs2155413	0.17
rs13091182	0.17
rs17400325	0.22
rs17428076	0.0031
rs6480859	0.11
chr14.54413001	0.041
rs4291789	0.72

p-values for significant SNPs for deviation from the proportional hazards assumption in the Cox model. For each SNP, we fit a Cox proportional hazards model including the SNP, sex, and five principal components as predictors, and then tested for independence of the scaled Schoenfeld residuals with time. Only one SNP deviates significantly from this assumption after correction for 22 tests. Plots for four example SNPs are shown in Figure S3.
